# COVID-19 and child and adolescent psychiatry: an unexpected blessing for part of our population?

**DOI:** 10.1007/s00787-020-01578-5

**Published:** 2020-07-04

**Authors:** Hilgo Bruining, Meike Bartels, Tinca J. C. Polderman, Arne Popma

**Affiliations:** 1 Department of Child and Adolescent Psychiatry, Amsterdam UMC, University of Amsterdam, Meibergdreef 5, 1105 AZ Amsterdam, The Netherlands; 2 Department of Biological Psychology, Vrije Universiteit Amsterdam, Amsterdam, The Netherlands; 3 Amsterdam Public Health Research Institute, Amsterdam UMC, Amsterdam, The Netherlands

The COVID-19 pandemic has left children and adolescents largely unaffected in terms of infectious morbidity and mortality [1]. A greater challenge for this age group is expected in dealing with lockdown and quarantine measures that may push children into crises and destabilize families [2, 3]. Especially, when quarantine measures are strict and in the presence of preexisting psychological or psychiatric vulnerabilities [4–6], a variety of negative outcomes are to be expected [7]. The COVID-19 outbreak has brought new challenges for child psychiatry and mental health services that must be addressed, including national guidelines covering interventions for major public health crises affecting children [8]. These threats and challenges have been rightfully addressed in several commentaries and are currently being studied across the globe [2].

Notwithstanding the importance of stressing the need for harm reduction in vulnerable children these days, here, we would like to draw attention to the flipside of the same coin. Clinical experience over the last weeks, as well as popular press coverage, shows that the sudden lockdown-induced changes for some children and families reduce daily stress, and sensory exposures and changes family routines. These changes seem to actually reduce child and adolescent mental illness symptoms and even improve well-being. Some kids seem to experience alleviation of social and sensory pressure and enjoy the more intensive family life. In this context, the crisis may provide a unique window of opportunity to test long-standing hypotheses on modern life stressors and mental health problems or psychiatric pathogenesis and well-being in developing children and adolescents [9, 10]. At no point in recent history, we have been given a similar chance to evaluate the effects of such a drastic environmental change; not only for the worse, but also for some for the good.

For instance, in The Netherlands and many other European countries, schools, companies, offices, social and sports clubs were all closed at once, but walks in the park and other outdoor activities were allowed under certain restriction. Nuclear family life was forcefully reinvented in many homes in sharp paradox with their usual busy normal routines of balancing work and family. Many parents became homebound schoolteachers whilst trying to keep their own professional lives going through online interactions. In the clinic, this led to mixed reports of being tied up in their houses whilst also being able to pay unprecedented amount of time and attention to each other. Indeed, another frequent noted observation was that certain patients and families seemed to thrive on the novel situation and context.

Therefore, we argue that the research agendas currently laid out to register and understand the negative effects of COVID-19 on child and adolescent (mental) health should also include the perspective of children and families who are benefitting from the societal changes.

Taking these heterogenous experiences in mind, we advocate an open scientific mind to COVID-19 studies by including ‘positive’ hypotheses and questions in addition to those testing negative expectations. We suggest that a diverse range of potential effects of the crisis, such as reduction of stress, improved sleep and relaxation, loss of social pressure, more time to think and improved affect. We caution against recruitment bias strategies when merely focusing on increased morbidity and problems. A wider approach will open up opportunities to go beyond studies on mental illness and mental health, by also including mental well-being. We should not only aim to help those who suffer, but also support well-being, which is a prerequisite for optimal psychological, social, and physical development. Research designs should, therefore, incorporate dimensional symptom evaluations and include multi-directional screenings of potential negative, but also positive influences. Hypotheses on both positive and negative responses of children and families to this crisis should be developed to understand the full breadth of impact on modern daily life routines and environments in future post COVID-19 times.

In all, the COVID-19 crisis confronts us with many novel realities and changes and has many insightful messages. For the field of child and adolescent psychiatry, and child development in general, these may be also related to learning how our social and economic environment interacts with child mental health and well-being. Comprehensive analysis of psychiatric morbidity in children and adolescents between the pre and post crisis times, in combination with carefully matched population-based control samples, offers an unprecedented window of opportunity to gauge how our current day society impacts well-being; for bad and for good.
